# Modulatory role of vitamins A, B3, C, D, and E on skin health, immunity, microbiome, and diseases

**DOI:** 10.1007/s43440-023-00520-1

**Published:** 2023-09-06

**Authors:** Mahika Joshi, Priyanka Hiremath, Jeena John, Niraja Ranadive, Krishnadas Nandakumar, Jayesh Mudgal

**Affiliations:** Department of Pharmacology, Manipal College of Pharmaceutical Sciences, Manipal Academy of Higher Education, Manipal, 576104 Karnataka India

**Keywords:** Skin diseases, Immunity, Vitamins, Microbiome

## Abstract

Disruption of the skin barrier and immunity has been associated with several skin diseases, namely atopic dermatitis (AD), psoriasis, and acne. Resident and non-resident immune cells and the barrier system of the skin are integral to innate immunity. Recent advances in understanding skin microbiota have opened the scope of further understanding the various communications between these microbiota and skin immune cells. Vitamins, being one of the important micronutrients, have been reported to exert antioxidant, anti-inflammatory, and anti-microbial effects. The immunomodulatory action of vitamins can halt the progression of skin diseases, and thus, understanding the immuno-pharmacology of these vitamins, especially for skin diseases can pave the way for their therapeutic potential. At the same time, molecular and cellular markers modulated with these vitamins and their derivatives need to be explored. The present review is focused on significant vitamins (vitamins A, B3, C, D, and E) consumed as nutritional supplements to discuss the outcomes and scope of studies related to skin immunity, health, and diseases.

**Graphical abstract:**

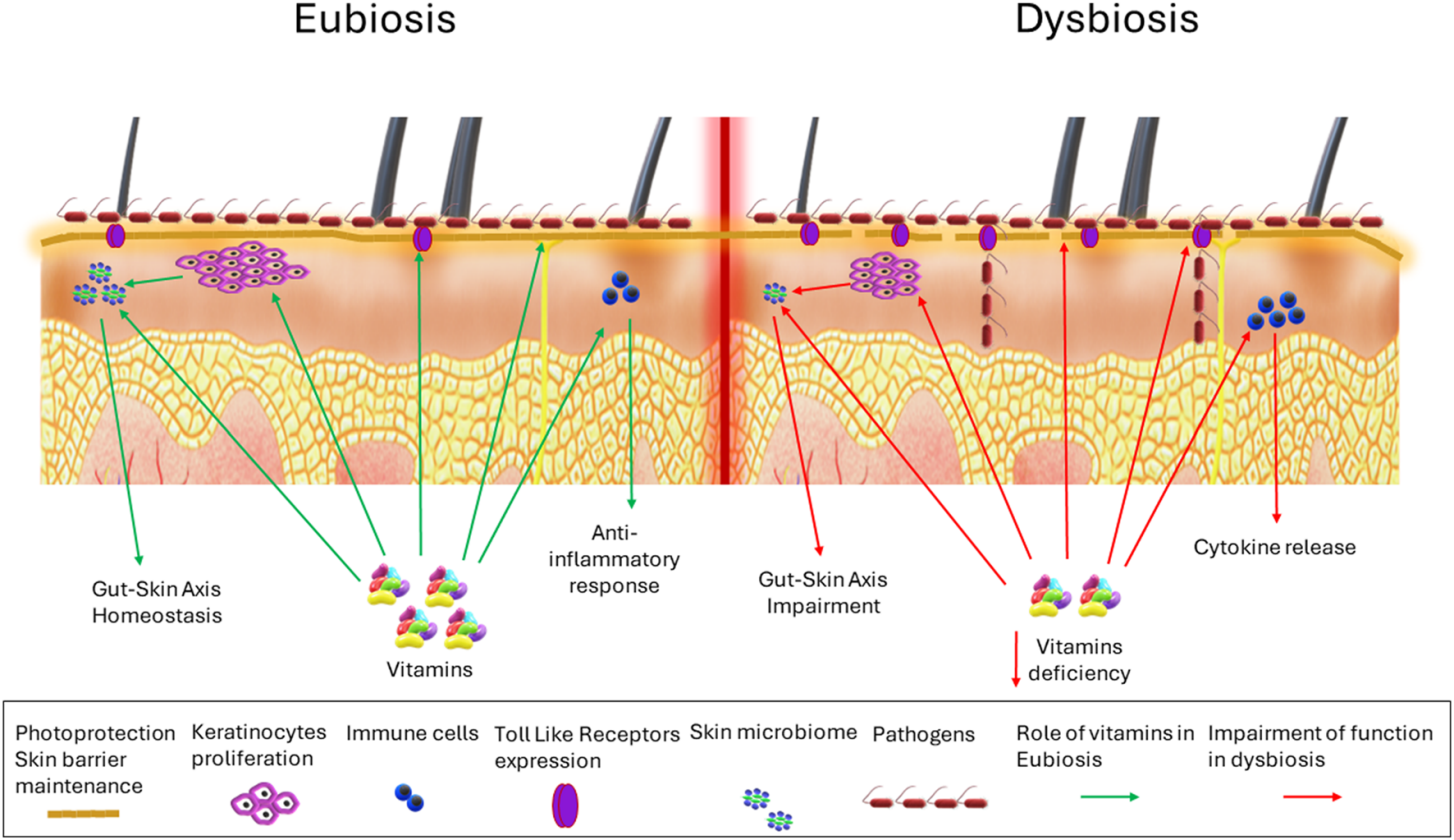

## Introduction

Skin, an essential organ of the integumentary system, acts as a defensive barrier between the internal organs and the environment. Its three layers (the epidermis, dermis, and hypodermis) that occupy a surface area of 2 m^2^ and approximately 15% of the total body mass, thus forming the most significant single organ in the human body. The skin serves several critical functions in the body, such as protection (against radiation, microorganisms, and mechanical, thermal, and chemical injuries), repair (responding to stress and altering its structure/composition during injuries), sensation (through mechanoreceptors, chemoreceptors, and nociceptors), thermoregulation (regulating body temperature of using insulation and sweating), synthesis of vitamin D, hormones and neurotransmitters, and excretion of water, uric acid, and ammonia [1–3]. The skin is constantly represented with information from environmental factors, including exposure to radiation, changes in temperature, relative humidity, and biological/chemical damage. The extent and the type of exposure determine the diversity in the structure and function of different parts of the skin. The mechanisms developed by the skin to maintain homeostasis and respond to external stimuli are widely distributed and highly regulated. This includes the response generated by the skin's immune system when exposed to biological insults/trauma or the synthesis of factors like parathyroid hormone-related protein (PTHrP), proopiomelanocortin-derived (POMC) β-endorphin peptides, corticotropin-releasing hormone (CRH) and urocortin peptides, catecholamines, and acetylcholine that respond to specific stimuli. Due to its size, location, and functional diversity, the skin plays a significant role in sending modulatory signals to the endocrine system that participate in a cascade of events necessary to maintain global and local homeostasis [2, 3].

The epidermis and the dermis layers of the skin consist of several types of immune-competent cells. Highly specialized epithelial cells are referred to as keratinocytes throughout the epidermis. They are periodically replaced by a single layer of basal keratinocytes, which proliferate constantly and give birth to cells that ascend toward the skin’s surface. Their maturation into corneocytes results in forming a protective barrier termed stratum corneum [4]. As a result of elastin and collagen fibers produced by fibroblasts, the dermis builds a thick extracellular matrix. Immune cells are engaged when pathogens are detected by blood capillaries irrigating the dermis and by lymphatic veins draining lymph fluid to lymph nodes. Moreover, the dermis includes hair follicles, oil glands, blood capillaries, and lymphatic vessels, which are the main conduit for immune cells like macrophages, dendritic cells, and T cells in the body [5]. The epidermis also contains a specialized subset of dendritic cells known as the Langerhans cells (LCs), which play a significant role in activating T cells in response to surface antigens [6]. Researchers found that the T cell responses are widely affected by cytokines secreted by keratinocytes. There is a range of different immune cells in the dermis that are implicated in allergic reactions in the skin, such as Natural Killer (NK) cells, eosinophils, monocytes, mast cells, and CD8+ cells. These immune cells, especially dendritic cells, and keratinocytes identify foreign particles or bacteria via Toll-like receptors, triggering subsequent immune responses, such as heightened production of AMPs and inflammatory cytokines. As a result, other immune cells, such as neutrophils, macrophages, and T cells, are attracted to the site of the immune response, resulting in an antibacterial action. Activated T cells in the skin eliminate infected keratinocytes to manage viral infections or release signals that attract other immune cells. After the virus is eliminated, lasting memory CD8+ T cells remain in the epidermis, ensuring immunity against future exposures [3, 7, 8]. However, it is found that commensal residents on the skin control the expression of AMPs and other various immune factors. AMPs in epithelial cells belong to several families of proteins, the main ones in the skin being cathelicidin and beta-defensin.

New metagenomics research indicates that the skin hosts a diverse range of microorganisms including bacteria, fungi, and viruses, all affected by factors like humidity, temperature, pH, and anti-microbial substances [9–11]. These commensal organisms habituated on the skin assist in wound healing and impede pathogen invasion by making anti-microbial peptides (AMPs) to prevent colonization, thereby suppressing the development of microorganisms’ biofilm [12, 13]. Eubiosis in the skin, i.e., the existence of a homeostatic balance of microbiota, plays a significant function in the maintenance of skin health. Thus, the skin has been demonstrated over time to play a considerable part in immunological function and not only as a physical barrier [14]. Epidermis, dermis, and subcutaneous fatty regions make up the skin’s layers. The skin also features hair follicles along with sebaceous, eccrine, and apocrine glands, which form separate niches housing a discrete microbiota [11]. The skin microbiota is mainly contributed by four members of phyla like *Actinobacteria*,* Firmicutes*,* Proteobacteria*,* and Bacteroidetes*. The most abundant and widespread genera that fall under the above category are *Propionibacterium*,* Corynebacterium*,* and Staphylococcus* [15]. In addition to genetic variables (such as genotype, age, and gender), external determinants, such as lifestyle, use of antibiotics, and cosmetics, can impact the variety and distribution of microorganisms on the skin [16]. In sebaceous glands, for example, lipophilic *Propionibacterium* species prevail, whereas *Staphylococcus* and *Corynebacterium* species are more prevalent in a wet environment. When it pertains to mycosis, *Malassezia* species are commonly encountered around the center of the body as well as in the underarm and feet. *Malassezia*, *Aspergillus*,* Cryptococcus* inhabit the plantar heel areas [9, 17, 18].

The skin microbiota further stimulates the expression of other highly complex host defense pathways, such as upgrading the levels of IL-1. This system comprises many proteins that react with each other, leading to the opsonization of the pathogens and triggering an inflammatory response which further facilitates their elimination [18]. The disruption between the homeostasis and microbiome may dysregulate some immune responses and cause skin disorders such as psoriasis or atopic dermatitis (AD) [16].

For sustaining healthy skin, a prominent corrective measure against the triggered immune response and free radicals-mediated damage is by the use of antioxidants. Antioxidants alter the signal transductions associated with skin damage [19]. Because of the natural antioxidant property of nutraceuticals, many individuals are augmenting their meals with multivitamins or isolated vitamin supplements, in addition to using topical skin care treatments on their faces. Vitamins A, B3, C, D, and E also exhibit anti-microbial effects through various pathways. For instance, vitamin C mitigates the proliferation of *Cutibacterium acnes* and increases barrier functionalities [20, 21]. Similarly, vitamin A is known to regulate mast cell function and support the treatment of various inflammatory disorders [22]. Hence, it is crucial to examine the impact of vitamins as antioxidants on skin immunity and their role in preventing dysbiosis. Thus, this article explores the various pathways in which vitamins A, B3, C, D, and E demonstrate their effects on skin immunity and prevent the growth of microbes that cause skin disorders.

## Methodology

A comprehensive review of publications, encompassing surveys and systematic reviews, was conducted to collectively analyze the roles played by vitamins A, B3, C, D, and E in skin immunity and various dermatological conditions. Studies were examined to elucidate the significance of the skin’s immune system and how it can be influenced by disruptions in the skin microbiome—a state called dysbiosis. Further, articles exploring the intricate pathways by which vitamins A, B3, C, D, and E contribute to fortifying skin immunity were discussed. Clinical trial data was examined to assess the efficacy of vitamins in mitigating inflammatory disorders triggered by the proliferation of microbiomes in conditions, such as AD, psoriasis, and chronic urticaria, among others. Scopus databases and PubMed were extensively used to identify articles around “skin immunity”, “skin microbiota”, and “skin immunity and vitamins A, B3, C, D, and E”. All the figures included in this manuscript were prepared using the Microsoft PowerPoint tools.

## Vitamin A

Retinoids are known for their beneficial effects in the prevention of various skin diseases. The class of retinoids, vitamin A, and its metabolites have been widely used in the cosmeceutical industry due to their beneficial effect in treating photo-damaged skin [23]. When vitamin A is consumed orally, the liver is responsible for converting dietary retinyl esters (RE) and beta-carotene to retinol. It is further esterified for storage or circulation within the body. Retinol binds to the retinol-binding protein and enters the capillaries in the dermis for distribution within the skin. The cellular uptake of retinol is mediated via endocytosis or specific receptors. Keratinocytes in human skin can convert retinol, a major form of vitamin A, to retinaldehyde and subsequently retinoic acid with the aid of dehydrogenases. Skin cells also could convert the precursor of vitamin A, beta-carotene to its subsequent metabolites. Cultures of human keratinocytes incubated with radiolabeled beta-carotene demonstrated increased concentrations of retinol. However, the percutaneous absorption of topically applied retinoids is low. Repeated applications of tretinoin in the form of creams, gels, or ointments have shown no significant changes in the plasma tretinoin concentrations and limited excretion through the urine and feces. Thus, minimal quantities of the active ingredient can enter the systemic circulation [24, 25].

**Fig. 1:**
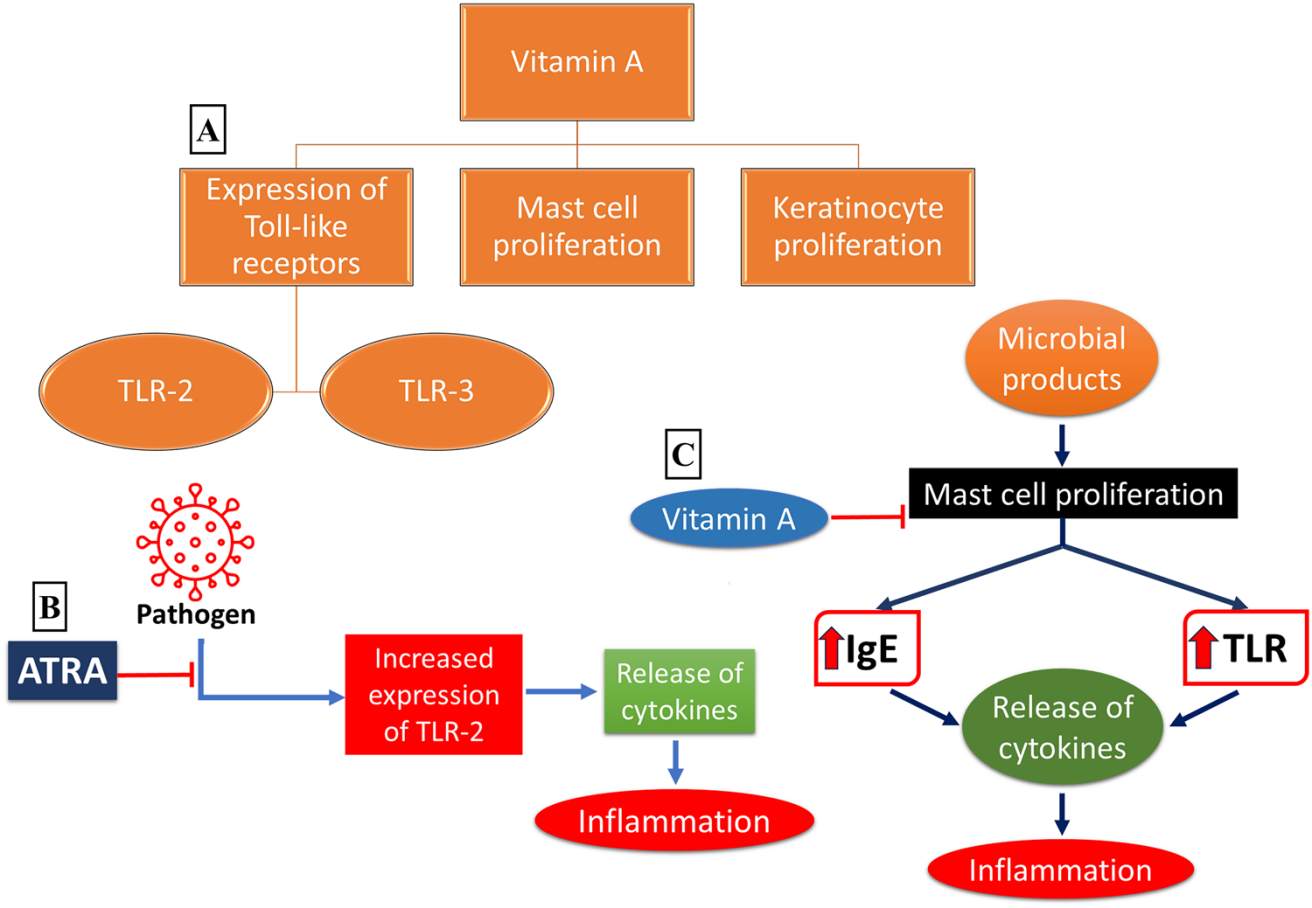
**a** Major pathways involved in the modulation of skin immunity by vitamin A [26]. **b** ATRA (All-trans-retinoic acid) downregulates the overexpressed TLRs activated by pathogenic bacteria and thereby inhibiting the release of cytokines, hence contributing to the anti-inflammatory effect, **c** vitamin A inhibits mast cell proliferation initiated by microbial products and thereby halts the process of IgE and TLR receptor activation with impaired release of cytokines and subsequently impedes inflammation. *TLR* Toll-like receptors, *IgE* immunoglobulin E

Vitamin A exhibits its effect on the skin’s innate immunity through three major pathways (Fig. 1). This primarily includes increased expression of Toll-like receptors-2 and 3 (TLR2 and TLR3), regulation of mast cells, and expression of anti-microbial proteins [26].

**Fig. 2:**
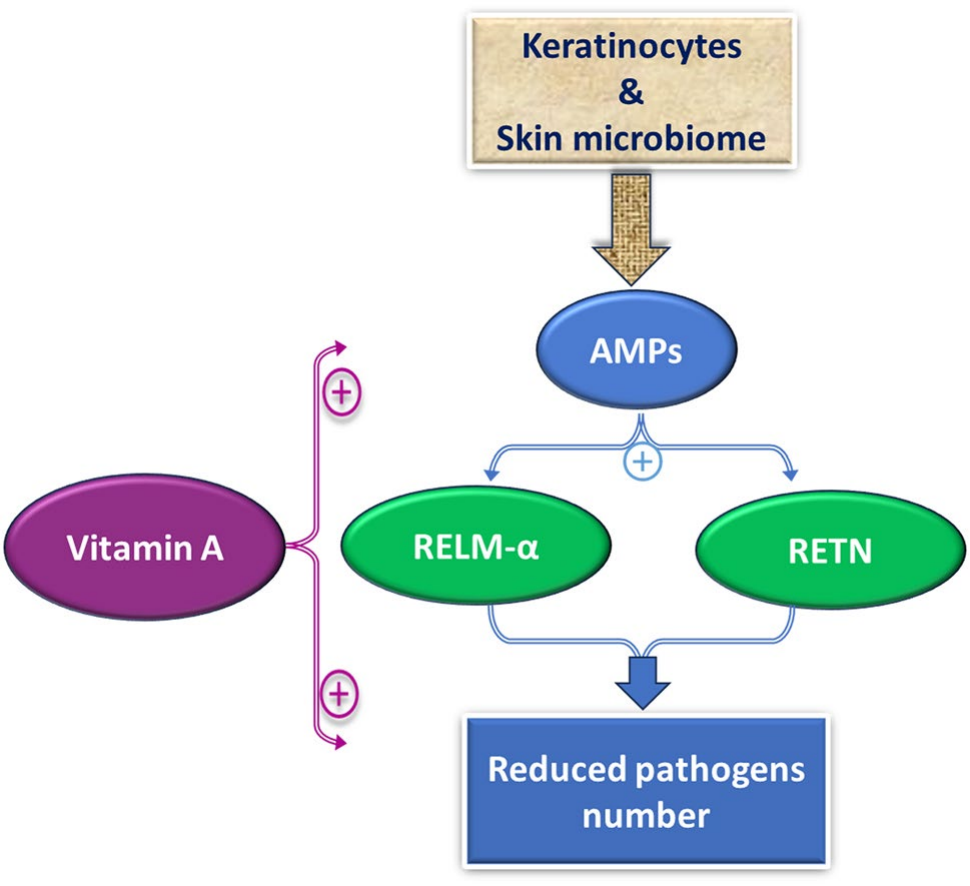
Vitamin A enhances the expression of anti-microbial peptides (AMPs) which are produced in response to skin bacteria along with keratinocytes and promotes host immunity. Vitamin A also activates Resistin-like molecule α (RELM-α) and Resistin (RETN) which in turn decreases the load of pathogenic bacteria

Toll-like receptors (TLRs) are important components of the skin's innate immunity [27]. Each TLR acts as a pathogen recognition receptor (PRR) that produces a pro-inflammatory response to pathogens or damaged cells. These are divided into different types based on their cellular localization [28, 29]. The activities of TLR2 and TLR3 are dependent on retinoic acid [26, 30, 31]. In a study conducted on human monocytes, *Cutibacterium acnes* was utilized to induce inflammation by increasing the expression of TLR2 and subsequently causing a release of cytokines. All-trans-retinoic acid (ATRA) demonstrated an anti-inflammatory response by downregulating TLR2 and its co-receptor CD14. Pre- and co-treatment of primary human monocytes with ATRA inhibited the function of TLR2 in triggering the release of monocyte cytokines (Fig. 2). ATRA also inhibited the induction of monocyte cytokines by *Cutibacterium acnes* [30].

Mast cells (MC) are known to play a significant role in the skin's innate immunity. These cells proliferate and get functionally altered in skin conditions, including AD, psoriasis, and chronic urticaria [32–34]. In response to microbial products, MC increases expression of TLRs and IgE receptors. This results in the secretion of pro-inflammatory cytokines [22]. Retinoic acid plays an intricate role in regulating mast cells and is widely used in the treatment of several inflammatory skin conditions (Fig. 1). A growing number of studies indicate that patients with AD are deficient in vitamin A and susceptible to bacterial colonization by *Staphylococcus aureus* [35–37]. It was found that by activation of MC, *Staphylococcus* δ-toxin potentially causes AD (40). MC releases cytokines including IL-4, IL-10, and IL-13 that can stimulate the conversion of CD4+ T cells to Th2 cells [38]. A supporting study illuminating the role of vitamin A showed decreased serum levels in infants with AD. Further, a vitamin A-deficient mice model projected a more severe Th2-mediated inflammation and exacerbated in vivo MC activation. The results also illustrated that supplementation of vitamin A could rescue its deficiency-mediated inflammation in AD by potentially supporting the homeostasis of MC [39]. Since T cells closely interact with MC and are modulated by the secretion of cytokines by these cells, retinoic acid may play a modulating role in mast cells through innate as well as adaptive immune processes [22].

Keratinocytes are involved in the production of AMPs in response to the skin microbiota. These proteins play a major role in regulating the bacterial communities on the skin and limiting the growth of pathogenic bacteria [40]. Recent findings have demonstrated that vitamin A can impact the expression of AMPs and thus promote host immunity against skin infections [26]. Bacterial species such as *S. aureus* can trigger the expression of resistin-like molecule α (RELMα), which is bactericidal in nature. Findings indicate that retinol promotes the expression of RELMα. In humans, a similar result was obtained where resistin (RETN) was found to be dependent on vitamin A for its expression. It was suggested that retinol enhances the expression of the RETN gene in sebocytes by the binding of retinoic acid receptors (RARs) to the RETN promoter [41]. This could help in explaining analogs of retinol, such as isotretinoin are commonly used in skin conditions like psoriasis and acne. Studies have demonstrated a decrease in the abundance of *Cutibacterium acnes* after the treatment of isotretinoin [42–44]. This shift in the microbial population could be attributed to the elevated expression of RETN in patients on isotretinoin (Fig. 2).

Vitamin A by virtue of its immunomodulatory effect protects against fungal infection due to *Candida albicans* [45]. In vitro and in vivo results from multiple trials reported the efficacy of vitamin A and its derivatives against a wide array of fungal infections in humans [46] for instance against *Aspergillus* spp. and *Microsporum* spp. Thus, vitamin A derivative such as retinoid can be explored in clinic as a potential therapeutic strategy against fungal infections.

## Vitamin B3

Niacinamide, alternatively known as nicotinamide, represents a water-soluble derivative of niacin, specifically belonging to the vitamin B3 group. With its diverse array of effects on the skin, niacinamide has gained considerable popularity as a constituent in cosmetic formulations. Notably, niacinamide is derived from nicotinic acid and is commonly referred to as vitamin PP due to its ability to counteract pellagra [22, 47].

Situated as the outermost layer of the skin, the epidermis plays a pivotal role in shielding the body against external agents, such as Ultraviolet radiation (UVR), pollution, and microorganisms. The delivery of vitamin B3 to the epidermis occurs through various mechanisms encompassing topical administration and oral supplementation. Upon topical application, vitamin B3 permeates the stratum corneum, which serves as the outermost layer of the epidermis, subsequently traversing to the viable epidermis [48]. Niacinamide participates in multiple metabolic processes and pathways (e. g. NAD synthesis) in the skin [49]. Once in the viable epidermis, vitamin B3 is converted to its active form, NAD^+^, which plays a critical role in energy metabolism and DNA repair [50].

Furthermore, the reduced forms of NAD^+^ like NADH and NADPH are also reported to have antioxidant properties. Dermo-cosmetics employ this vitamin for its anti-inflammatory, anti-microbial, and barrier as well as for its photo-protective properties [47]. However, much of the current research has been focused on the ability of niacinamide to fight inflammation and acne. Niacinamide crosses the stratum corneum more efficiently than nicotinic acid and has a high tolerability profile as it does not provoke skin irritation or redness [51].

The anti-inflammatory properties of niacinamide are linked to its ability to suppress the expression of poly-(ADP-ribose) polymerase-1 (PARP-1) enzyme that governs the nuclear factor kappa B (NFκB) transcription [52]. These gene transcriptions are regulated by PARP-1 in various immune cells, including dendritic cells, macrophages, and lymphocytes. As a result of PARP-1 inhibition, pro-inflammatory cytokine levels decline [53]. This makes it beneficial in the treatment of skin diseases, such as acne vulgaris and other skin disorders. Acne vulgaris, is a skin condition of different etiologies and is identified by the occurrence of both inflammatory and non-inflammatory skin lesions. *Cutibacterium acnes* plays a significant part in the progression of this disease by triggering the release of pro-inflammatory cytokines such as interleukin-8 (IL-8) through the stimulation of toll-like receptors (TLR 2) [54]. IL-8 is a neutrophil-specific target interleukin derived from keratinocytes, enhances its growth, and triggers sebum formation. It has been established that acne lesions cause the stimulation of transcription factors, resulting in the release of PARP-1 [55]. Research shows that on topical application of 2% nicotinamide, there was a decrease in sebum production in a Japanese experimental group and a drop in the levels of sebum on the skin surface in a Caucasian experimental group [56]. Other studies conducted in controlled clinical demonstrated that 4% niacinamide was proven to be as efficient as 1% clindamycin in patients with moderate acne [57]. Furthermore, in vitro investigations revealed that nicotinamide exhibits its anti-inflammatory function by suppressing leukocytes and their cellular response and reducing the release of IL-8, a cytokine-induced in response to *Cutibacterium acnes* [32].

Apart from acne, nicotinamide has been tested against AD, which is a multifaceted chronic inflammatory skin condition due to several genetic and environmental factors. It progresses further because of genetic alterations in the structural protein called filaggrin [58]. A drop in filaggrin levels leads to skin barrier deficiencies, thus causing more significant trans-epidermal water loss, further aggravating the skin’s vulnerability to environmental allergens and pathogenic organisms, resulting in chronic skin irritation [59]. During clinical studies, it was observed that there was an increase in the *Staphylococcus aureus* bacterium population in individuals affected by AD, which led to an overall reduction in the variety of skin microflora [60, 61]. Inflammatory and immunological responses elicited by this dysbiosis include a decline in the number of circulating T cells, impaired functioning of TLRs, and an increase in CD4+ cells. Interleukins like IL-4, IL-5, and IL-13 are secreted by these cells, which stimulate IgE synthesis [60]. A clinical study was undertaken for eight weeks to investigate the moisturizing impact of nicotinamide in patients with atopic dry skin and it was confirmed that surface application of niacinamide helps to sustain the skin barrier, decreasing trans-epidermal water loss (TEWL) and increasing the production of skin proteins and ceramides [62].

Psoriasis is an autoimmune disease analogous to AD, but it is distinguished by neutrophil accumulation and elevated nitric oxide levels [63]. As previously stated, nicotinamide is an anti-inflammatory agent. It can inhibit immunological responses in conjunction with the nitric oxide synthase enzyme [64]. When used with calcipotriol in the treatment of psoriasis, nicotinamide proved to be an effective adjuvant [65]. Additionally, it was also demonstrated that topical administration of 0.25% 1-methylnicotinamide helped to treat rosacea, another dermatological condition, after four weeks of clinical testing [66].

1-methyl nicotinamide(1-MNA) and nicotinamide-N-oxide are the two primary metabolites of nicotinamide. 1-MNA undergoes further metabolism to produce 1-methyl-2-pyridone-5-carboxamide and 1-methyl-4-pyridone-5-carboxamide. Despite having multiple therapeutic mechanisms, 1-MNA’s anti-inflammatory properties appear to be its primary benefit. Additionally, 1-MNA is one of the analogs of nicotinamide adenine dinucleotide (NAD^+^) [67, 68].

In non-melanoma skin cancer, vitamin B3 especially nicotinamide was found to be useful in modulates skin immune response and showed chemo-protective effects [69]. Nicotinamide, when taken orally, not only prevents skin cancer but also reduces the financial burden of treatment costs [70].

## Vitamin C

Vitamin C is integral in ameliorating skin pathologies, including acne, psoriasis, progressive purpura, or allergic contact dermatitis [71]. It is often used as a part of formulations that provide an anti-inflammatory effect. In the case of acne, *Cutibacterium acnes* triggers pro-inflammatory mediators, which leads to the generation of acne through its involvement in the skin keratinocytes and sebaceous glands of the pilosebaceous follicle [21]. A combination of vitamin C, zinc, and clarithromycin was found to render an antibacterial effect on *Cutibacterium acnes *in vitro [72].

Vitamin C is found in both the layers of the skin, the dermis, and the epidermis, with the latter having higher concentrations [73, 74]. UVR or pollutants may affect the levels of vitamin C in the epidermis layer by causing degradation [75–77]. When consumed orally, vitamin C is transported from the bloodstream to the layers of the skin through specific transporter proteins [78]. Keratinocytes have a larger capacity for transporting vitamin C in comparison to the epidermis which has limited vascularization [78, 79]. Vitamin C can also be topically administered. Applying vitamin C topically can provide benefits to the skin, but the outermost layer of skin, called the stratum corneum, can limit its absorption [80]. Removing this layer through various methods can improve absorption [81]. Studies on laboratory animals suggest that vitamin C absorption depends on pH, with a pH below 4.0 promoting absorption. Concentrations of up to 30% have been tested, but 20% is the most effective for absorption. Ascorbic acid can cross the epidermis and reach deeper layers of the skin. However, vitamin C can degrade over time due to exposure to air, heat, and/or light [80]. Stable synthetic derivatives, such as ascorbate phosphate and ascorbyl palmitate, have limited permeation, and absorption and may have toxic effects [82]. Adding other antioxidant compounds can increase the stability of topical vitamin C solutions [83]. Human studies have reported no adverse effects from using solutions containing 0.6–10.0% vitamin C or its synthetic derivatives [84].

**Fig. 3:**
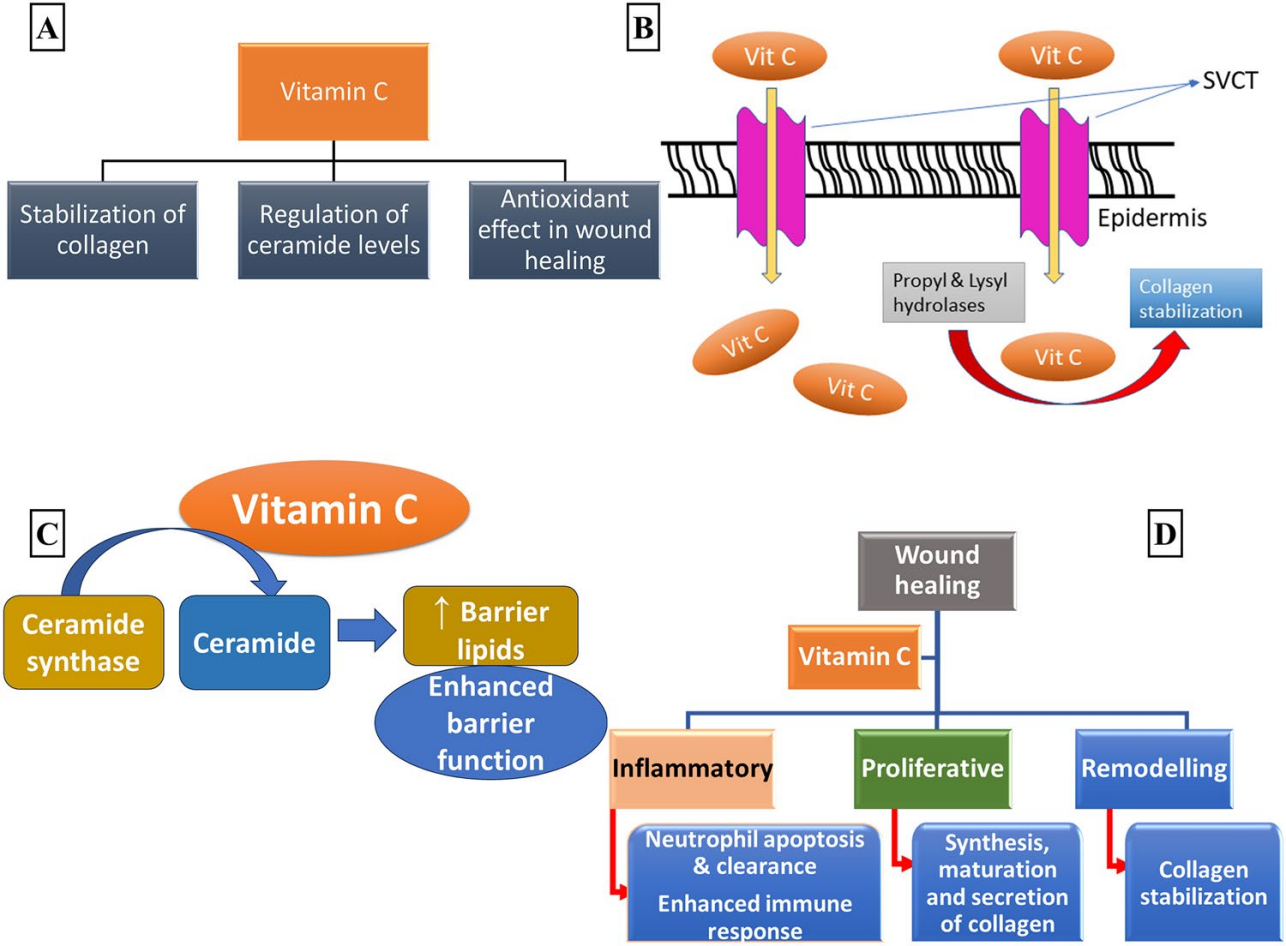
**a** Role of vitamin C in the maintenance of skin health; **b** role of vitamin C (Vit C) in collagen stabilization. The accumulation of Vit C in the dermis and epidermis takes place through sodium-dependent vitamin C transporter (SVCT) which is then responsible for serving as a co-factor for propyl and lysyl hydrolases to stabilize collagen [78]; **c** Role of vitamin C in the regulation of ceramide levels. Vitamin C acts as a co-factor in the synthesis of ceramide by the conversion of ceramide synthase into ceramide. Ceramide enhances the level of barrier lipids and plays an important role in maintaining the structure and permeability barrier function of the skin. **d** Role of vitamin C in the three stages of wound healing

Vitamin C is reported to exert its therapeutic effect by three mechanisms, (1) collagen stabilization, (2) ceramide regulation, and (3) wound healing effect (Fig. 3a). Two sodium-dependent vitamin C transporter (SVCT), isoforms 1 and 2 helps in the entry and accumulation of vitamin C in the dermis and the epidermis [78]. This suggests that vitamin C plays a crucial role in skin health (Fig. 3b). Deficiency in vitamin C results in diseases like scurvy characterized by bleeding gums and poor wound healing [85, 86]. Such individuals are more prone to infections since they have a down-regulated immune system [87]. Vitamin C acts as a co-factor for the propyl and lysyl hydrolase enzymes that provide stability to the tertiary structure of collagen [88]. Hence, a deficiency in vitamin C is a plausible reason for the symptoms experienced by patients with scurvy.

Studies have demonstrated the role of vitamin C in skin inflammation [89]. One such study indicated decreased plasma levels of vitamin C (6–31 μmol/L) in patients with AD. Further, an inverse relationship between the epidermal ceramide levels and plasma vitamin C levels was also observed in the patients [90]. Ceramide is an essential component of the stratum corneum and its increased production occurs due to the stimulation of ceramide synthase [40]. So, vitamin C act as a co-factor for enzymes involved in collagen synthesis as well as for the enzyme ceramide synthase. Vitamin C supplementation in a keratinocyte culture results in increased differentiation and barrier functions through the synthetic and biosynthetic pathways [20]. This points toward the vital role of vitamin C in enhancing the synthesis of barrier lipids (Fig. 3c).

Vitamin C is known to substantiate the process of wound healing (Fig. 3d). It enhances the expression of mediators that promote wound healing and causes a decline in the pro-inflammatory mediators [91]. The inflammatory process is initiated by neutrophils that migrate to the site of infection (also called chemotaxis), to release anti-microbial proteins and reactive oxygen species (ROS) [92]. Ultimately, they undergo apoptosis and are cleared away by macrophages (Fig. 3d). Vitamin C influences all three stages of inflammation, including chemotaxis, phagocytosis, killing the microbiota, apoptosis, and clearance of neutrophils [87].

Primarily, vitamin C is accumulated in the neutrophils through SVCT2 (sodium-dependent vitamin C transporter 2), and its oxidized form (dehydroascorbate or DHA) is transported through glucose transporters (GLUT) [93, 94]. Vitamin C levels within the cells are rapidly increased to 10 mM through the reduction of DHA to ascorbate. The accumulation of vitamin C in the neutrophils can play a protective role in oxidative damage and aid in regenerating antioxidants such as glutathione that are present in the membrane [94]. It also attenuates the generation of oxidants that activate ​​pro-inflammatory transcription factor nuclear factor κB (NFκB) [95, 96]. Patients with conditions such as chronic granulomatous disease (CGD), an immunodeficiency disease where the neutrophils and macrophages are functionally defective, are left susceptible to bacterial and fungal infections [97]. They may develop skin infections due to bacteria such as *Serratia marcescens* or *Staphylococcus aureus* and fungal infections due to *Aspergillus* species [98]. Studies have indicated decreased infections and an improvement in leukocyte chemotaxis through supplementation of vitamin C [98, 99]. This could be attributed to its effect on microtubule assembly [100].

Vitamin C also supports the process of apoptosis of neutrophils. During this process, enzymes such as caspases play a critical role in the marking and clearance by macrophages [101]. However, caspases are sensitive to the ROS generated by neutrophils and are likely to get inactivated due to the activation of the neutrophils [102, 103]. In vitro studies conducted on human neutrophils demonstrate that vitamin C could potentially play a protective role in the oxidant-sensitive caspase-dependent apoptotic process induced by *Escherichia coli* [104]. Supporting evidence shows a decrease in apoptosis and persistence of inflammatory loci by isolated neutrophils of vitamin C-deficient Gulo mice [105, 106].

## Vitamin D

Vitamin D, also called calciferol, is a vital fat-soluble nutrient extensively known for its role in bone health and calcium homeostasis. There are multiple ways to administer vitamin D, including the following: the most common method is taking oral supplements in the form of capsules, tablets, or liquids. Sun exposure can also help the skin produce vitamin D, but the amount produced depends on factors, such as skin color, time of day, season, and location. Vitamin D can also be injected into the muscle or applied topically as creams, ointments, or lotions. A nasal spray can also deliver vitamin D, which is absorbed through the nasal mucosa [107, 108].

UVR serves as a natural source of radiation that encompasses a range of effects on human health, both advantageous and detrimental. Its impact extends to various aspects of cutaneous and systemic homeostasis, including the synthesis of vitamin D, regulation of skin pigmentation, enhancement of the skin's barrier function, modulation of the immune system, regulation of blood pressure, and even mood regulation [109]. However, undue exposure of the skin to solar UVR can result in several forms of damage, including sunburn, photo-aging, and the development of skin cancer. Consequently, it is strongly advised to safeguard the skin against UVR by adopting multiple protective measures, such as the diligent use of sunscreen. Nevertheless, the recommendation of stringent Ultraviolet B (UVB) protection, as advocated by several guidelines, has triggered a debate due to its potential association with vitamin D deficiency [110]. To compensate for reduced solar exposure, the option of incorporating low-dose vitamin D dietary supplementation has been proposed. The overlapping action spectra of both harmful and beneficial effects prompt inquiries into the rationale behind the long-standing evolutionary process of gene-environment interactions. Nonetheless, the synthesis of pre-vitamin D3 does not necessitate prolonged exposure to UVB radiation, and excessive UVB irradiation can lead to sunburn, consequently, compelling individuals to limit or avoid sun exposure [111]. Thus, maintaining a delicate equilibrium between the advantageous and detrimental consequences of UVR is crucial. Ongoing efforts are focused on developing optimized sunscreens that facilitate vitamin D synthesis while minimizing the risk of erythema [112].

Upon exposure of the skin to UVB radiation, a crucial process unfolds whereby pre-vitamin D3 is synthesized from 7-dehydrocholesterol (7-DHC), primarily occurring within the keratinocytes situated in the stratum basale and stratum spinosum layers of the epidermis. Subsequently, this pre-vitamin D3 is converted into its active form, vitamin D3, and facilitated into the systemic circulation with the aid of a binding protein [113]. Notably, alternative photoproducts, namely tachysterol3, and lumisterol3, can also arise from the conversion of pre-vitamin D3. However, these photoproducts exhibit biological inactivity and exhibit limited entry into the circulation, thereby serving as a protective mechanism against potential vitamin D toxicity. While it is acknowledged that vitamin D3 can undergo further degradation into additional photoproducts upon sunlight exposure, the precise biological significance of these resultant photoproducts remains ambiguous [114]. According to Dr. Michael Holick, a prudent approach to sun exposure entails selectively exposing arms and legs to midday sunlight for brief intervals twice a week, potentially meeting the individual's vitamin D requirements. Nevertheless, it is important to recognize that various factors, such as seasonal variations, geographical latitude, skin pigmentation, advancing age, and the use of sunscreen, possess the capacity to influence the synthesis of vitamin D within the skin, thereby significantly impacting overall vitamin D levels in the body [115].

There are multiple ways to activate vitamin D that differ from the conventional pathway. These alternative methods can still lead to the production of active vitamin D [113]. One of these alternative methods involves a particular enzyme called CYP11A1, which is present in various tissues including the skin and adrenal gland. Unlike the classical pathway, the activation of vitamin D through this enzyme is not reliant on the liver and kidneys [116]. Another alternative pathway of vitamin D activation involves the conversion of vitamin D to calcidiol by enzymes CYP2R1 and CYP27A1, found in different tissues throughout the body. From calcidiol, calcitriol (the active form of vitamin D) can be produced directly, bypassing the need for the kidneys to activate it in the classical pathway. The significance of these alternative pathways in vitamin D metabolism is still being investigated, and therefore their importance is not fully understood [117, 118]. Studies conducted by Bubshait et al. indicated that the transdermal route of vitamin D is potentially safe and can give desired results to raise vitamin D levels [119].

Keratinocytes residing in the epidermis possess the necessary enzymatic machinery to facilitate the conversion of vitamin D into its biologically active form. Moreover, these keratinocytes also express the vitamin D receptor (VDR), which acts as a regulatory factor in gene expression [120]. Vitamin D is predominantly available in two primary forms: ergocalciferol (vitamin D2), derived from plant sources, and cholecalciferol (vitamin D3), obtained from animal sources. The precursor molecule, 7-dehydrocholesterol, abundantly present in keratinocytes, undergoes a non-enzymatic breakdown upon exposure to ultraviolet (UV) light, resulting in the formation of pre-vitamin D3, specifically cholecalciferol. This molecule is subsequently subject to further enzymatic processing to yield the active metabolite of vitamin D3, known as 1,25-hydroxyvitamin D, or calcitriol [121]. The interaction between calcitriol and the VDR assumes a crucial role in the immunological system of the skin. Studies conducted in the past have indicated that calcitriol can enhance cellular calcium concentration, thereby promoting the activation of structural proteins and ultimately facilitating keratinocyte differentiation [122]. Furthermore, this ligand–receptor interaction holds significance in the development of the epidermal barrier, as it contributes to the synthesis of ceramides, vital components involved in maintaining the integrity and functionality of the skin's barrier function [123–125].

On the other hand, calcitriol binds to the TLR2 receptor and CD14, which changes the levels of anti-microbial peptides, such as human beta-defensins (HBDs) and cathelicidin via CYP27B1 induction [123]. In recent years, researchers have been more intrigued by the preponderant effect of vitamin D on immune cells. Immunological functions of vitamin D include the inhibition of T lymphocyte cells and the induction of T-reg (regulatory T cell) [126, 127]. On T-lymphocyte membranes, vitamin D activates the expression of G protein-coupled receptor 2, which is engaged in the T cell-mediated pathway during skin inflammation, culminating in its retention in the epidermal cells [128, 129].

Several studies have documented that vitamin D insufficiency may have a main function in the development of psoriasis. This chronic skin disease is identified by Th-17 cells and their associated immuno-stimulatory cytokines (IL-17, IL-21, IL-22), including tumor necrosis factor-alpha and atypical AMP response, resulting in its overproduction ultimately triggering the inflammation and proliferation of keratinocytes, leading to the development and persistence of psoriatic lesions [130]. Vitamin D therapy has been shown to reduce the activity of TLR2 and TLR4 in monocytes [131]. The active form of calcitriol exerts its anti-inflammatory activity by suppressing pro-inflammatory cytokines and HBDs and slowing down the chemical responses of dendritic cells, which are abundant in psoriatic lesions [131, 132]. Decreased VDR mRNA activity was found to result in increased cathelicidin LL-37 expression in psoriatic lesions [133].

Previous reports have shown that treating patients with psoriatic skin with an isoform A of VDR yielded better results [124]. The VDR gene is responsible for regulating the effects of vitamin D and various intracellular signaling pathways that are involved in cell differentiation. The A-allele of this gene has been found to bind more efficiently to the Cdx-2 protein and has increased transcription activity compared to the G allele. Genetic variations in the VDR gene can affect vitamin D synthesis, metabolism, and degradation, and it is expressed in various organs, including the intestine, thyroid, and kidneys, where it plays a crucial role in calcium homeostasis [134]. VDRs can repress the expression of 1alpha-hydroxylase, which is responsible for activating 1,25(OH)2D3, and it can induce the expression of the 1,25(OH)2D3 inactivating enzyme CYP24. The VDR is also expressed in keratinocytes, and calcitriol, which is a natural ligand for VDR, can inhibit the proliferation and induce differentiation of human keratinocytes [134, 135].

Unlike psoriasis, the epithelial layer in AD contains less cathelicidin, making it more vulnerable to infection by *S. aureus*. One of the studies showed that macrophages activate TLRs, leading to increased expression of VDR and its genes, which ultimately leads to increased production of cathelicidin, thereby reducing susceptibility to bacterial infections [136]. In vitro*,* evidence reveals that vitamin D has a functional role in acne vulgaris. However, according to recent data, it was indicated that 1,25-hydroxyvitamin (1,25 OH2D) lowers the proliferation of Th-17 cells whose release was triggered by *Cutibacterium acnes* during the progression of acne lesions [137].

## Vitamin E

Vitamin E, an essential fat-soluble vitamin, is present in the form of eight compounds, namely α-, β-, γ- and δ-tocopherols and tocotrienols [138]. Despite its lower concentrations in the cell membranes, it is the first line of defense against damage caused by free radicals on cell membranes. It is considered a significant antioxidant in tissues and can prevent lipid peroxidation by quenching free radicals. The deficiency of vitamin E can result in reduced immune function [139].

Within the skin, the epidermis exhibits higher concentrations of vitamin E compared to the dermis [74]. Specifically, α-tocopherol emerges as the prevailing form of vitamin E observed in the skin of individuals who have not undergone vitamin E supplementation. Nevertheless, detectable quantities of γ-tocopherol [140], as well as other tocopherols and tocotrienols derived from the diet [141], may also be present in the skin.

Following its initial accumulation in the sebaceous glands, vitamin E is subsequently transported to the skin surface via sebum [142, 143]. It should be noted that oral ingestion of vitamin E requires a minimum of seven days to cause noticeable changes in the vitamin E content of sebum [142, 144]. While the skin lacks specific transport proteins exclusively dedicated to vitamin E, its lipophilic properties enable it to penetrate all underlying layers of the skin [145]. Exposure to UV light [140, 146, 147] or ozone [143, 148, 149] can diminish the levels of vitamin E in the skin, particularly within the stratum corneum. This reduction in vitamin E content may be attributed to the destructive effects of these environmental factors on the antioxidant properties of vitamin E. Furthermore, it has been observed that vitamin E levels in the human epidermis decline with age [74], potentially due to the altered structure of the aging epidermis, which facilitates increased penetration of UV light into this layer [150].

Much like the transportation of vitamin E to the stratum corneum via sebum, the topical usage of vitamin E can infiltrate both the epidermis and dermis [147, 148]. Nevertheless, our comprehension of the rate at which vitamin E is absorbed through the skin and the elements affecting its permeation in humans remains considerably unclear. This is due to the varied employment of concentrations and timeframes in diverse studies. Generally, it’s believed that even solutions containing a minimal 0.1% concentration of vitamin E can enhance its cutaneous levels [151]. Notably, following topical application, the concentration of vitamin E in the dermis significantly increases, with sebaceous glands likely serving as a major site of accumulation [152]. Nonetheless, the concentration of vitamin E in the dermis remains lower than that observed in the stratum corneum. Skin that primarily relies on dietary vitamin E typically contains α- and γ-tocopherol [140, 144, 145]. In contrast, skin exposed to synthetic vitamin E topically may encompass a mixture of various tocopherols and/or tocotrienols [147, 148]. Upon topical application, vitamin E tends to accumulate in the cell membranes and extracellular lipid matrix of the stratum corneum, thereby contributing to antioxidant defense mechanisms. However, a significant portion of topically applied vitamin E is susceptible to degradation in the skin upon exposure to UV light [147], indicating its inherent instability and tendency for loss from the skin. Thus, enhancing the stability of topical vitamin E formulations becomes crucial. One approach to improve the stability of topical vitamin E solutions involves the use of vitamin E conjugates, commercially produced esters of tocopherol that exhibit resistance to oxidation while retaining the ability to penetrate the layers of the skin. However, it is important to note that vitamin E conjugates do not possess antioxidant functions, and the efficacy of these formulations can vary considerably depending on the specific compound and the model system utilized [153].

**Fig. 4:**
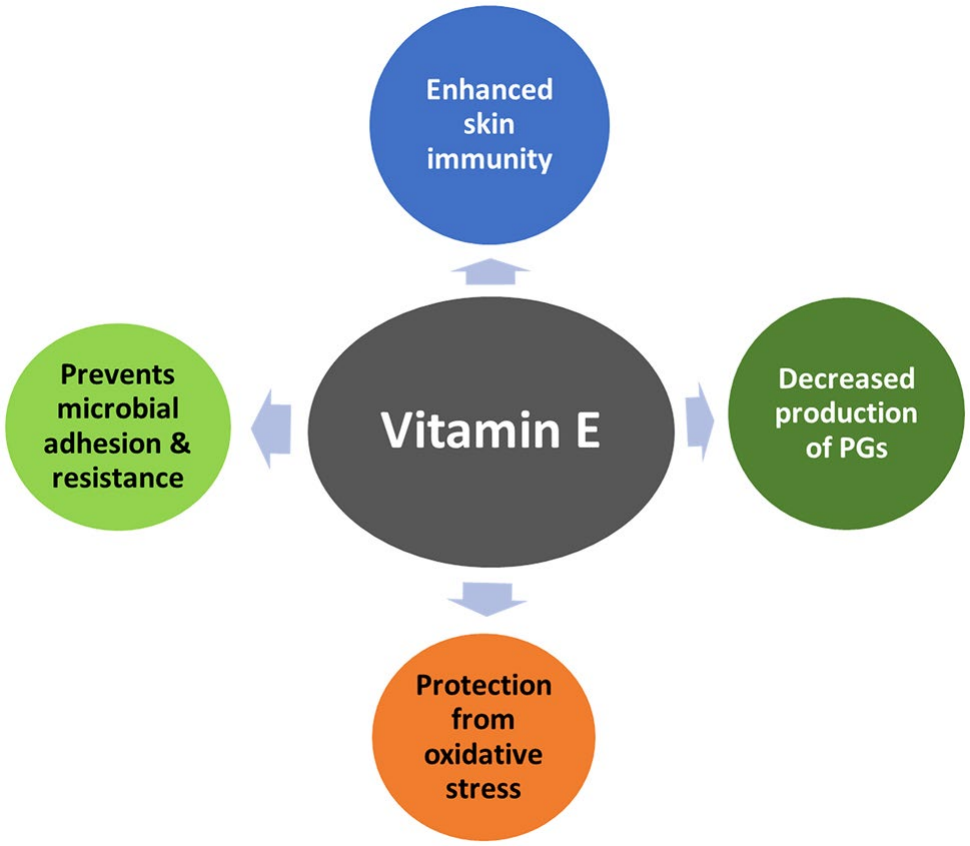
Anti-inflammatory and immune-boosting properties of Vitamin E in maintaining skin health

Vitamin E is known to render an increased immune response through two mechanisms (Fig. 4). First, due to its antioxidant properties, it can protect the cell membranes of macrophages from oxidative stress. Second, it plays a role in curbing the production of prostaglandins [154, 155]. A study on AD patients demonstrated an inverse relationship between serum vitamin E and IgE levels in the treatment group. The evaluation groups showed improvement in conditions associated with AD, like facial erythema or lichenification after supplementation with vitamin E [156]. Since enhanced production of IgE is thought to be a significant factor in the development of AD, these findings suggest the role vitamin E can play in improving AD symptoms. Similarly, various studies dealing with the effect of Vitamin E in managing AD have demonstrated a negative association between serum IgE levels and alpha-TP, thus supporting the theory mentioned earlier [157–159].

In acne vulgaris, where the colonization of *Cutibacterium acnes* is the known causative organism [160], the production of ROS and lipid peroxides (LPO) is a part of the progression of the disease. The oxygen acquires unpaired electrons during the generation of ROS and forms free radicals that cause lipid peroxidation and cytokine production. This ultimately results in inflammation, and the skin remains exposed to chronic oxidative stress [161, 162]. Additionally, the generation of oxidants during lipid peroxidation can also rupture the follicular walls. Studies have indicated a negative correlation between serum vitamin E levels and the severity of acne vulgaris [163]. Supplementation of vitamin E in patients with acne vulgaris showed positive results in reducing the severity of acne for eight weeks. Further, there was a decrease in the number of inflammatory and non-inflammatory lesions two weeks into the treatment. These findings establish the role of vitamin E in neutralizing oxidants generated during inflammatory conditions like acne vulgaris [162].

Tocotrienol was found to inhibit the production of nitric oxide, prostaglandin E2 (PGE2), cytokines tumor necrosis factor-α, interleukin (IL)-4, IL-8, induced nitric oxide synthase (iNOS), cyclooxygenase (COX)-2 and NF-ĸB expression in THP-1 monocytes challenged with *Candida albicans* [164, 165]. THP-1 monocytes are involved in the innate immune response to fungal infections and found that the cell wall components of *Candida* enhance the secretion of TNF-alpha, IL-8, and IL-1β. Vitamin E is shown to downregulate the pathways involved in the release of IL-1α, IL-1β, IL-10, IL-8, IL-6, and IL-12. Further, it also plays a role in suppressing the expression of cytokines, such as interferons and TNF-α, which are produced by fibroblasts in response to the fungal virulence factors [165].

An interesting study conducted on prosthetics also highlighted the role of vitamin E in decreased septic failure caused by implants. The ultra-high molecular weight polyethylene (UHMWPE) components used in prosthetics can be a source of bacterial infections due to the physicochemical interactions between the material and the microbe [166]. Since microbial adhesion is the primary step in the pathogenesis of the disease, vitamin E stabilized with UHMWPE (VEPE) can play a role in decreasing bacterial adhesion [167]. The VEPE showed increased resistance toward *Staphylococcus epidermis*, *Staphylococcus aureus*, *Escherichia coli*, and *Candida albicans*, thus inciting further research on extrapolating the antioxidant effect of vitamin E in modulating skin microbial adhesion and ultimately preventing diseases [166–168].

## Gut–skin axis and vitamins

The potential role of the gut microbiome has evolved as an emerging topic in the research fraternity, particularly the gut–brain axis, the gut–lung axis, and the gut–skin axis. The analogous nature of gut and skin in terms of functionality and purpose confirms their potential role in associated pathological conditions. Innervated and vascularized nature, epithelial cells, AMPs, phagocytes in the innate immune system, and higher cellular turnover rate are the common characteristics shared by both gut and skin tissues [188]. Modulation in the immune system is responsible for establishing the link between the gut and skin homeostasis because of their capability to host a wide range of microorganisms to balance with the human system. So, disturbance in the balance may lead to impairment in the gut–skin axis [189]. Vitiligo is a type of skin disease characterized by the loss of melanocytes with the appearance of patchy white skin. A direct correlation between gut and skin was proved by developing a preclinical model of vitiligo where the mice have been subjected to antibiotic-induced depletion of gut microbiota, which resulted in skin depigmentation [190]. This was also supported by the clinical evidence where vitiligo patients found gut dysbiosis with reduced *Bacteroides* compared to the healthy controls [191].

Different studies have reported the role of gut microbes in skin inflammatory diseases by regulating the immune system [192, 193]. The IL-23/IL-17 inflammatory pathway in psoriasis is reported to be regulated by both skin and gut microbiota [194]. Administration of different probiotics like *Bifidobacterium infantis*, *Lactobacillus pentosus* was found to have beneficial effects in psoriatic patients. The same was also reported in the imiquimod-induced psoriasis model of mice. This was mainly evidenced by the reduction in various pro-inflammatory mediators like TNF-α and IL-6 by probiotics [195, 196]. Similarly, gut dysbiosis manifested by the decrease in microbiome diversity was observed in acne vulgaris patients [197], and probiotics consumption prevented the occurrence of AD in different patient populations [198]. Apart from these, skin pathological conditions like rosacea [199], dandruff, and seborrheic dermatitis [200] were also associated with gut dysbiosis, which was then ameliorated by administering the administration of probiotics.

As the gut microbiome serves as the source of different vitamins, gut dysbiosis might alter their levels, contributing to skin dysbiosis [201]. However, there is a dearth of evidence on the role of vitamins modulating the gut–skin axis. Therefore, vitamin’s impact on the gut–skin axis needs to be studied for establishing their plausible connect in skin disorders. The occurrence of microbiota in the gut and skin sharing common characteristics and functions may enable us to understand critical microorganisms and their mechanisms of action responsible for each skin disorder.

## Conclusion

The skin serves as a crucial organ for innate immunity. Disruption of this barrier and immune function has been linked to various skin diseases, such as AD, psoriasis, and acne. Recent advancements in understanding the skin microbiota have shed light on the intricate communication between these microbiota and skin immune cells. Vitamins, known for their antioxidant, anti-inflammatory, and anti-microbial properties, have emerged as potential modulators of immune signals and hold promise for improving skin health and managing skin diseases (Table 1). Further, the status of a few clinical trials has been listed (Table 2), which indicates the ongoing testing of these vitamins for the treatment of skin diseases. Thus, examining the potential of specific vitamins (A, B3, C, D, and E) when consumed as drug/nutritional supplements will highlight their future therapeutic/preventing potential in the studies related to skin immunity, health, and diseases.

**Table 1:** Summarized role of vitamins in maintaining skin immunity and health with their mode of action in different dermatological conditions

Vitamin	Mode of action	Skin diseases
Vitamin A	Keratinocyte proliferation and differentiation, mast cell proliferation, expression of TLRs and AMPs	Acne vulgaris, AD, psoriasis
Vitamin B3	Antioxidant, antibacterial, anti-inflammatory, and photo-protective effects	AD, acne vulgaris, psoriasis, rosacea
Vitamin C	Anti-inflammatory and antioxidant effects, collagen stabilization, and ceramide synthesis	Acne vulgaris, photo-aging
Vitamin D	Keratinocyte differentiation, antibacterial, anti-inflammatory	Psoriasis, acne vulgaris
Vitamin E	Skin immunity, antioxidant, antibacterial, and anti-inflammatory effects	AD, acne vulgaris

**Table 2:** Clinical trials investigating the effects of vitamins A, B3, C, D, and E on skin conditions

S. no.	Name of intervention	Skin condition/disease	Status/outcome	References
1	Niacinamide (4%) and virgin coconut oil (30%) moisturizing cream	Contact dermatitis of hands	Not completed	[169]
2	Topical application of nicotinamide + calcipotriol	Psoriasis	Additional benefits to psoriasis patients	[170]
3	Niacinamide (4%) and desonide (0.05%)	Hyperpigmentation	Significant depigmenting improvement versus placebo	[171]
4	Evaluation of acceptability, skin barrier restoration, and balance of atopic skin using moisturizer	AD	Not completed	[172]
5	Nicotinamide + antibacterial adhesive + zinc-pyrrolidone carboxylic acid cream	Acne vulgaris	Reduced lesions	[173]
6	Retinyl palmitate-loaded topical ethosomes	Facial acne vulgaris	Effective in controlling acne and tolerable to skin	[174]
7	Topical vitamin A (all-trans retinol) cream	Skin aging	Improvement in natural aging associated fine wrinkles	[175]
8	Topical vitamin A with or without azithromycin	Acne vulgaris	Not completed	[176]
9	Micro-needling with topical vitamin C	Acne scars of acne vulgaris	Ineffective outcome	[177]
10	Ascorbic acid versus diode laser	Gingival hyper-pigmentation	Not completed	[178]
11	Vitamin c injection versus the conventional surgical depigmentation	Melanin hyper-pigmentation	Comparable results with both techniques	[179]
12	Topical tranexamic acid and topical vitamin C with microneedling	Facial melasma	Effective and safe outcomes	[180]
13	Glycolic acid (70%) peel with vitamin C	Acne scars	Well-tolerated treatment of acne scarring in Asian skin	[181]
14	Combination of phototherapy and oral vitamin D	Vitiligo autoimmune disease skin	Not accessible	[182]
15	Blue light (453 nm) treatment over three months compared to vitamin D	Psoriasis vulgaris	Completed	[183]
16	Vitamin D neoadjuvant with photodynamic therapy (PDT)	Actinic keratosis	Improved PDT efficiency clinically	[184]
17	Active vitamin D	Acne vulgaris	Improvement of the clinical status of acne patients	[137]
18	Bioactivity of vitamin D in the skin after oral supplementation	Healthy, no evidence of skin disease	Variability in vitamin D receptor expression	[185]
19	Oral lactoferrin with vitamin E and zinc	Acne vulgaris	Significant reduction in acne lesions	[186]
20	Oral vitamin E	AD	Improved symptoms	[187]

This review may serve as a navigational tool for keen researchers to gain exhaustive knowledge. By elucidating the effects of these vitamins on skin immunity, new therapeutic avenues can be explored for enhancing the understanding of the intricate interplay between vitamins, the skin microbiome, and immune responses. Although the present review dealt with specific vitamins and their role in skin health, the impact and contribution of vitamins toward skin homeostasis is enormous. The limitation of the present review includes a dearth of direct evidence on the vitamins’ role in gut–skin axis. The review is also limited to a few major vitamins i.e., vitamins A, B3, C, D, and E. The vitamin dosage schedules, their bioavailability, the factors influencing skin microbiome, and their pro/anti-microbial properties can also be reviewed and is the limitation of this review. Thus, there is a vast scope of reviewing pharmacological and dermatological drug development considering the vitamins’ impact in skin diseases and the gut–skin axis.

## Future perspectives

Counteracting the generation of free radicals by acting as antioxidants makes vitamins an important part of skin immunity. Vitamins exert a wide range of biological functions in maintaining skin health and support the endogenous defense system in combating pathological conditions. The increased uptake of vitamins in recent times for boosting immunity formed an integral part of the human diet whose absence could significantly hamper biological functions. The bidirectional link between the changes in gut microenvironment and skin immunity in the development of different dermatological conditions may introduce therapeutic strategies that could balance gut–skin homeostasis. Even though different vitamins are proven to be efficacious in various skin pathologies, the involvement of these substances and their isoforms in gut microbiota in improving the alterations need to be explored. This could probably evolve as a mechanistic pathway that can link various aspects of skin immunity and gut microbiome that eventually identifies new treatment avenues and reducing stigmatization in people with skin disorders.

## Data Availability

Data sharing does not apply to this review article.

## Abbreviations

AD: Atopic dermatitis
AMP: Anti-microbial peptides
ATRA: All-trans-retinoic acid
CGD: Chronic granulomatous disease
COX: Cyclooxygenase
CRH: Corticotrophin
GLUT: Glucose transporters
HBD: Human beta-defensins
IgE: Immunoglobulin E
IL: Interleukin
iNOS: Inducible nitric oxide synthase
IU: International unit
LPO: Lipid peroxides
MC: Mast cells
NAD: Nicotinamide adenine dinucleotide
NFκB: Nuclear factor kappa B
NK cells: Natural killer cells
PARP-1: Poly-(ADP-ribose) polymerase-1
POMC: Proopiomelanocortin
PTHrP: Parathyroid hormone-related protein
RAR: Retinoic acid receptors
RELMα: Resistin-like molecule α
RETN: Resistin
ROS: Reactive oxygen species
SVCT: Sodium-dependent Vitamin C transporter
TEWL: Trans-epidermal water loss
TLR: Toll-like receptors
TNF-α: Tumor necrosis factor-alpha
UHMWPE: Ultra-high molecular weight polyethene
VDR: Vitamin D receptor
